# Integration of transcriptomic data reveals lipid metabolic heterogeneity and identifies GSTO1 as a therapeutic target in acute myeloid leukemia

**DOI:** 10.3389/fimmu.2026.1815163

**Published:** 2026-06-30

**Authors:** Fangmin Zhong, Zihao Wang, Jialin Huang, Linfeng Jin, Fangyi Yao, Xiaozhong Wang

**Affiliations:** Jiangxi Province Key Laboratory of Immunology and Inflammation, Jiangxi Provincial Clinical Research Center for Laboratory Medicine, Department of Clinical Laboratory, The Second Affiliated Hospital, Jiangxi Medical College, Nanchang University, Nanchang, Jiangxi, China

**Keywords:** acute myeloid leukemia, GSTO1, immunotherapy, lipid metabolism, prognostic signature, tumor microenvironment

## Abstract

**Background:**

Acute myeloid leukemia (AML) is an aggressive hematologic malignancy with poor prognosis and significant heterogeneity. Lipid metabolic reprogramming is a key hallmark of cancer, yet its systemic characterization and clinical relevance in AML remain largely unexplored.

**Methods:**

Multi-omics data were integrated, including one single-cell RNA-seq dataset and bulk transcriptomes from nine AML cohorts. Lipid metabolism activity was assessed using GSVA. Consensus clustering based on lipid metabolism pathways identified molecular subtypes. A lipid metabolism-related prognostic signature (LMRS) was constructed via machine learning algorithms and validated across nine independent cohorts. Functional validation was performed in AML cell lines using GSTO1 inhibition.

**Results:**

Single-cell analysis revealed significant upregulation of lipid metabolism pathways in AML malignant cells, particularly in progenitor-like subpopulations. Three lipid metabolism-based subtypes (C1–C3) were identified, with the C3 subtype exhibiting the highest metabolic activity, an immunosuppressive microenvironment, and the worst prognosis. A robust nine-gene LMRS model was developed, which effectively stratified patients into high- and low-risk groups with distinct survival outcomes. LMRS demonstrated superior predictive accuracy over existing models, was independently prognostic, and correlated with chemotherapy and immunotherapy resistance. Inhibition of GSTO1 significantly induced apoptosis and ROS production in AML cells.

**Conclusion:**

This study comprehensively defines lipid metabolic heterogeneity in AML, establishes a clinically applicable prognostic signature, and underscores lipid metabolism as a key driver of AML progression and immunosuppression. Targeting lipid metabolism, particularly through GSTO1 inhibition, represents a promising therapeutic strategy.

## Background

Acute myeloid leukemia (AML) is a highly heterogeneous hematological malignancy characterized by abnormal proliferation and differentiation arrest of myeloid precursor cells (1). Despite certain advancements in treatment modalities such as chemotherapy and hematopoietic stem cell transplantation, the prognosis for AML patients remains poor, especially for elderly patients and those with adverse genetic features, with unsatisfactory 5-year overall survival rates (2). Therefore, in-depth exploration of the molecular mechanisms of AML and identification of new prognostic markers and therapeutic targets are highly important for improving patient outcomes.

In recent years, metabolic reprogramming has emerged as a focal point in tumor research (3). Tumor cells remodel their metabolic networks to meet the demands of rapid proliferation for bioenergetics and biosynthesis (4, 5). Lipid metabolism, a crucial component of cellular metabolism, not only participates in cell membrane construction and energy storage but also plays a key role in signal transduction, apoptosis, and immune regulation (6, 7). Numerous studies have demonstrated that lipid metabolism abnormalities are closely associated with the occurrence, development, and treatment resistance of various solid tumors (8–10). However, systematic research on lipid metabolism in AML is still relatively limited, and its specific mechanisms of action and clinical significance in AML cells remain unclear.

This study aims to systematically analyze the lipid metabolic characteristics of AML cells by integrating multiomics data, identify molecular subtypes related to lipid metabolism, and construct a prognostic model based on lipid metabolism-related genes (LMRGs) to predict patient prognosis and treatment response (11). We further validated the biological roles of key LMRGs in AML cells through functional experiments, providing a new theoretical basis and potential strategies for the precise classification and targeted therapy of AML.

## Methods

### Data acquisition and preprocessing

**Figure 1:**
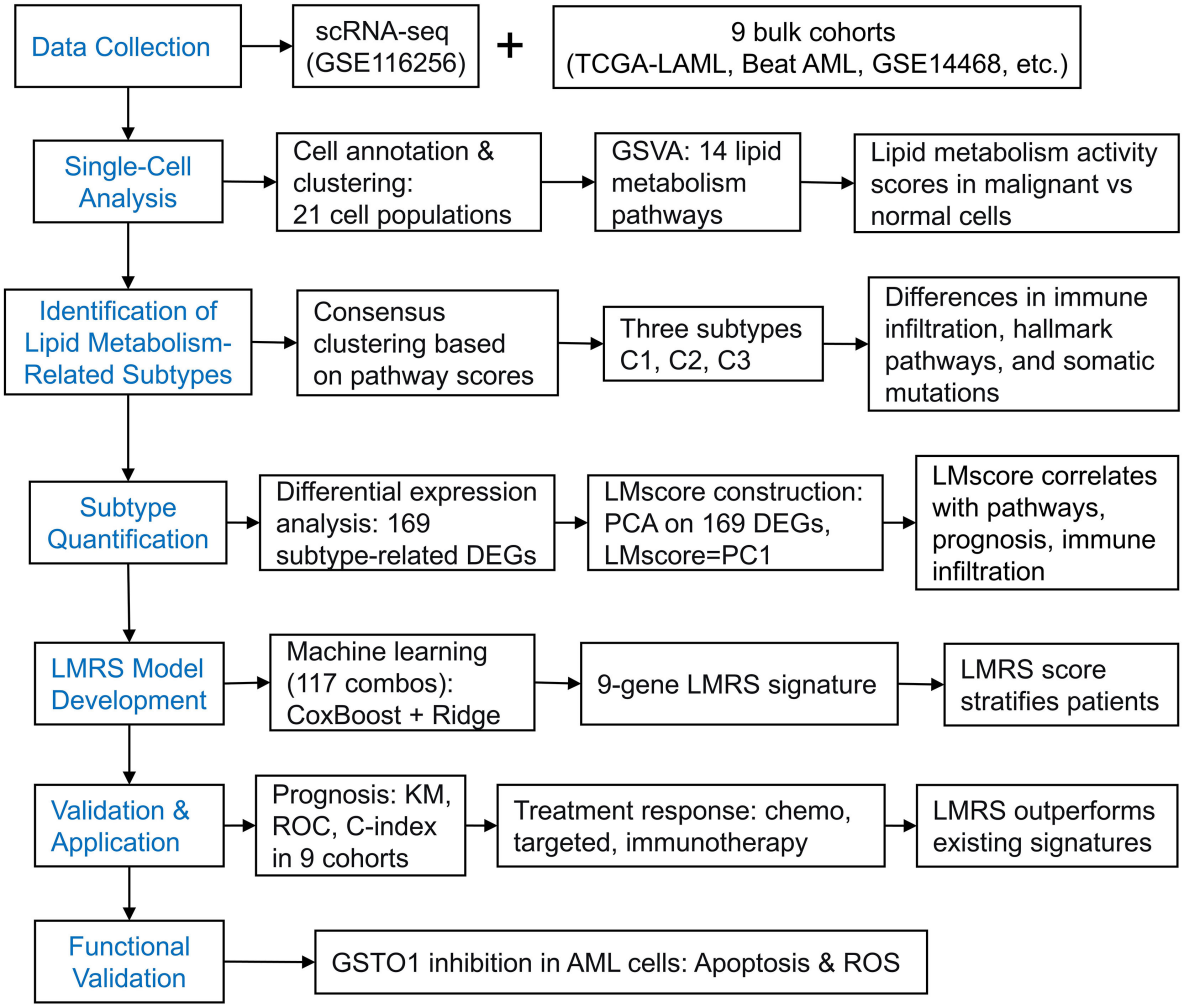
The overall analytical workflow.

The overall analytical workflow is summarized in **Figure 1**. Gene expression data and corresponding clinical information were obtained from multiple public databases. These cohorts included the TCGA-LAML cohort from the UCSC Xena database (https://xena.ucsc.edu/) and eight Gene Expression Omnibus (GEO) datasets, namely, GSE10358-GPL570, GSE12417-GPL96, GSE12417-GPL570, GSE37642-GPL96, GSE37642-GPL570, GSE71014-GPL10558, GSE14468-GPL570, and Beat AML. The dataset IMvigor210 for validating the immune therapy response was also obtained from public resources (http://research-pub.gene.com/IMvigor210CoreBiologies/). All expression data were uniformly preprocessed, including gene annotation and log2 transformation. Somatic mutation data were obtained from the TCGA database (https://portal.gdc.cancer.gov/). A summary of all cohorts, including their sources, platforms, sample sizes, clinical contexts, and specific roles is provided in **Supplementary Table 1**. The IMvigor210 cohort is a non−AML external cohort (urothelial carcinoma) used only for hypothesis−generating immunotherapy response analysis.

### Single-cell data analysis and lipid metabolism activity scoring

Single-cell RNA sequencing (scRNA-seq) data were obtained from the GEO database (GSE116256). Standard quality control, normalization, dimensionality reduction (PCA), and cell clustering were performed via the Seurat R package. The cell subpopulations were annotated on the basis of the markers and methodology described in the published literature (12), ultimately identifying 6 AML malignant cell subpopulations and 15 normal cell subpopulations. Lipid metabolism pathway gene sets were downloaded from the KEGG database. The gene set variation analysis (GSVA) algorithm was used to score the activity of 14 lipid metabolism pathways for each cell subpopulation (13). To ensure the robustness of our findings, we validated the lipid metabolism pathway activity scores obtained from GSVA using the single-sample Gene Set Enrichment Analysis (ssGSEA) algorithm as an alternative method.

### Bulk data deconvolution and correlation analysis

Using the CIBERSORTx online tool, deconvolution analysis was performed on batch transcriptome data such as TCGA-LAML data, with the scRNA-seq data annotated in this study as the feature reference matrix, to estimate the relative proportions of 21 cell subpopulations in each patient sample (14). The Spearman correlation coefficient between cell proportions and GSVA lipid metabolism pathway scores was subsequently calculated.

### Consensus clustering identified lipid metabolism subtypes

On the basis of the GSVA scores of 14 lipid metabolism pathways in the TCGA-LAML cohort, unsupervised consensus clustering analysis was performed on AML patients via the ConsensusClusterPlus R package (15). The clustering parameters were set as follows: the maximum number of clusters k = 9, and 1000 repeated samplings. The stability of the clustering results was verified through principal component analysis (PCA) visualization. Finally, k = 3 was selected as the number of clusters, and the patients were divided into three lipid metabolism-related subtypes (C1, C2, and C3).

### Differential expression and functional enrichment analysis

Differential expression gene (DEG) analysis among the three lipid metabolism subtypes (C1 vs C2 vs C3) was conducted via the limma R package. Genes were selected on the basis of a false discovery rate (FDR) < 0.01. Gene Ontology (GO) and Kyoto Encyclopedia of Genes and Genomes (KEGG) pathway enrichment analyses were performed on the obtained DEGs via the clusterProfiler R package, with an FDR < 0.05 as the significant enrichment criterion.

### Construction of the lipid metabolism score

On the basis of the expression matrix of DEGs related to lipid metabolism subtypes, dimensionality reduction was performed via the PCA algorithm. PCA was performed using the prcomp function in R (stats package, version 4.3.0) with center = TRUE and scale = TRUE. The first principal component (PC1) was extracted and used as a comprehensive score of lipid metabolism activity for each sample and was named the LMscore. For external validation cohorts, the same scaling parameters (mean and standard deviation) and PC1 loadings derived from the TCGA−LAML training cohort were applied to calculate LMscore values.

### Construction and validation of the lipid metabolism-related prognostic signature model

In nine AML training cohorts, univariate Cox regression analysis was performed on LMRGs to screen for genes significantly associated with overall survival (OS) (P < 0.05) in at least five AML cohorts. The selected prognostic-related LMRGs were subsequently incorporated into an integrated modeling framework, and various algorithm combinations (including stepwise Cox, CoxBoost, LASSO, ridge regression, etc., a total of 117 combinations) were tested. Prognostic models were constructed in each training cohort and validated in other cohorts. The average C-index was used to evaluate the predictive performance of each model in all the validation cohorts. The optimal model was selected, and the lipid metabolism risk score for each patient was calculated. Patients were divided into high-risk and low-risk groups on the basis of the best cut-off value of LMRS. The prognostic predictive performance and independence of the LMRS were evaluated in multiple independent validation cohorts through Kaplan–Meier survival analysis, receiver operating characteristic (ROC) curve analysis, and univariate and multivariate Cox regression analyses.

The final LMRS score was calculated as a linear combination of the expression levels of the 9 genes weighted by their ridge regression coefficients. The formula is:


$$LMRS=\;\sum_{1}^{i}(Coefi*ExpGenei)\;$$

The complete coefficient table for score calculation can be found in **Supplementary Table 2**.

### Predictive analysis of treatment response

In the cohort containing treatment response information (GSE14468, Beat AML), the differences in LMRS between the response group and the nonresponse group were compared, and the ROC curve was plotted to evaluate the ability of the LMRS to predict the response to chemotherapy. Using the drug sensitivity data of the Beat AML cohort, the correlation between the LMR and drug AUC values was analyzed. In the IMvigor210 cohort, the relationships between LMR and immune treatment response and prognosis were evaluated.

### Cell lines and *in vitro* functional validation

Human AML cell lines (NB4 and MOLM13) were purchased from the American Type Culture Collection (ATCC). The cells were treated with a GSTO1 inhibitor (GSTO1-IN-1). GSTO1-IN-1 (MedChemExpress, USA) was dissolved in DMSO. Cells were treated with serial dilutions of GSTO1-IN-1 (0, 1.25, 2.5, 5, 10, 20, 40 μM) for 48 hours. Cell proliferation was detected via a CCK-8 assay, and the half-maximal inhibitory concentration (IC50) was calculated using nonlinear regression in GraphPad Prism. For CCK-8 assays, cell viability was normalized to DMSO-treated controls (set as 100%). All experiments were performed with three independent biological replicates (n=3). Apoptosis and ROS assays were performed at a concentration of 5 μM for 24 hours. The apoptosis rate was detected via an Annexin V-FITC/PI apoptosis detection kit via flow cytometry. Intracellular reactive oxygen species (ROS) levels were detected with a DCFH-DA probe via flow cytometry.

### Statistical analysis

All the statistical analyses were performed via R software. Differences between two or more groups were analyzed using the Wilcoxon rank sum test and the Kruskal–Wallis test, respectively. Survival analysis was performed via the Kaplan–Meier method and log-rank test. Correlation analysis was conducted via Spearman correlation analysis. Adjusted P values were applied for DEG analysis and pathway enrichment; for drug sensitivity and other exploratory analyses, uncorrected P values are reported and annotated as exploratory. All the statistical tests were two-sided, and P < 0.05 was considered statistically significant.

## Results

### Assessment of lipid metabolism activity in AML cells

**Figure 2:**
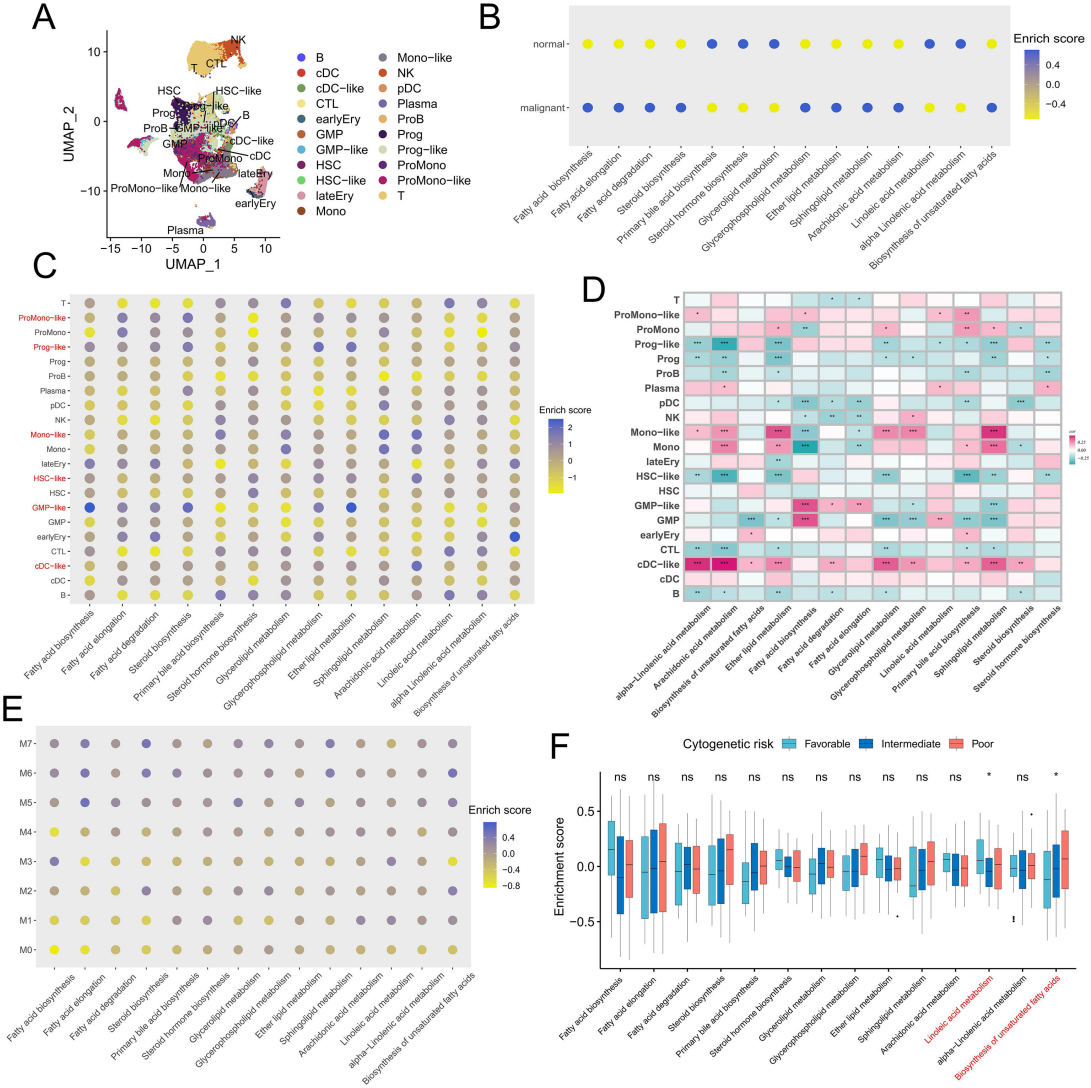
Characteristic lipid metabolism scores of acute myeloid leukemia (AML) cells. **(A)** Classification features of each cell subpopulation in the AML single-cell dataset. **(B)** Differences in lipid metabolism scores between AML malignant cells and normal cells. **(C)** Comparison of lipid metabolism scores among different cell types. **(D)** Correlation analysis of the proportions of different immune cell infiltrates within AML samples and lipid metabolism scores in the TCGA-LAML cohort. **(E, F)** Differences in lipid metabolism scores among patients with different FAB classifications **(E)** and cytogenetic risk stratifications **(F)** in the TCGA-LAML cohort. (ns, no significance; *P<0.05; **P<0.01; ***P<0.001).

We first evaluated the lipid metabolism characteristics of AML cells at the single-cell level. By classifying the cell subpopulations in the AML samples, a total of 21 cell populations were identified, including six types of AML malignant cells (cDC-like, GMP-like, HSC-like, Mono-like, Prog-like, and ProMono-like) and 15 types of normal cells (B cells, T cells, NK cells, etc.) (**Figure 2A**). The GSVA algorithm was subsequently used to score the activity of 14 lipid metabolism pathways. The results revealed that the activity of fatty acid metabolism-related pathways was significantly increased in AML malignant cells, especially in ProMono-like, Prog-like, and GMP-like cells (**Figure 2B**). In addition, the overall activity of metabolic pathways such as glycerophospholipid, ether lipid, sphingolipid, and arachidonic acid pathways also increased in AML malignant cells, further indicating that AML cells have more active lipid metabolism characteristics (**Figure 2C**). We subsequently performed deconvolution analysis of the relative proportions of 21 AML cell types in the TCGA-LAML cohort. Correlation analysis revealed that the proportions of cDC-like and Mono-like cells were significantly positively correlated with the activity of most lipid metabolism pathways, whereas the proportion of GMP-like cells was strongly correlated with the activity of the fatty acid metabolism pathway (**Figure 2D**). The results of FAB subtype analysis indicated that the overall activity of lipid metabolism pathways was greater in patients with M5- to M7-type AML (**Figure 2E**). Although there were no significant differences in pathway activity among the different cytogenetic risk groups, the activity of the unsaturated fatty acid biosynthesis pathway gradually increased with increasing risk level (**Figure 2F**).

### Identification of lipid metabolism-related molecular subtypes

**Figure 3:**
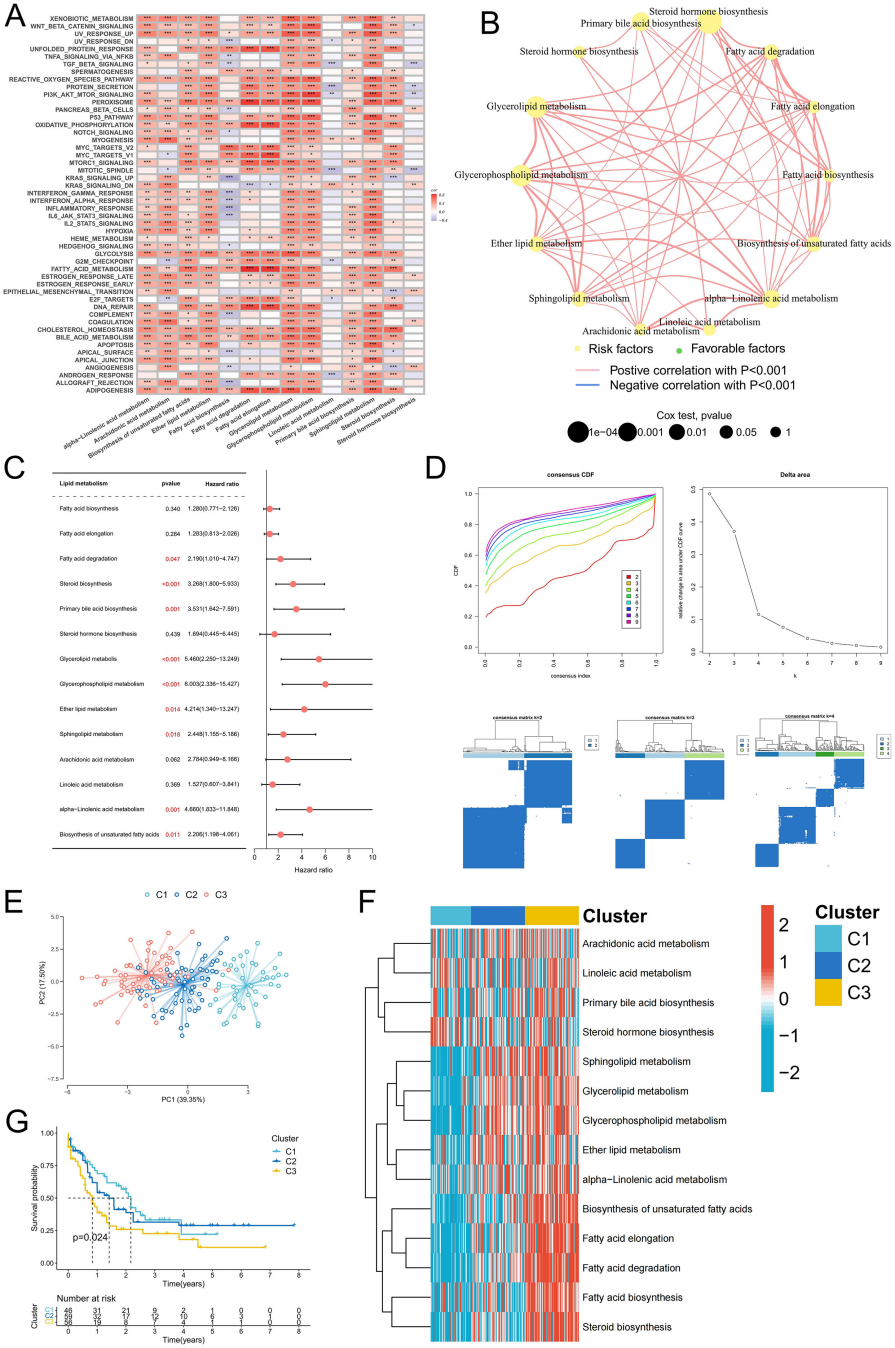
Identification of lipid metabolism-related molecular subtypes. **(A)** Correlation analysis between the lipid metabolism score and tumor hallmark pathway activity score. **(B)** Intercorrelations among lipid metabolism pathways and their associations with patient prognosis. **(C)** Results of univariate Cox regression analysis of lipid metabolism pathways. **(D)** Identification of lipid metabolism-related molecular subtypes on the basis of a consensus clustering algorithm using lipid metabolism pathway scores. **(E)** Validation of the stability and reliability of the clustering results via principal component analysis (PCA). **(F, G)** Differences in the lipid metabolism score (The darker the red color, the higher the pathway enrichment score; the darker the blue color, the lower the pathway enrichment score.) **(F)** and overall survival (OS) of patients **(G)** according to different molecular subtypes. (*P<0.05; **P<0.01; ***P<0.001).

Firstly, we confirmed in the TCGA-LAML cohort that the enrichment scores of lipid metabolism pathways calculated by the ssGSEA algorithm and the GSVA algorithm were significantly positively correlated, demonstrating the robustness of these methods (**Supplementary Figure 1**). We further evaluated the clinical relevance of lipid metabolism pathways. The results revealed that the activity of lipid metabolism pathways was significantly positively correlated with most cancer hallmark pathways, suggesting that these pathways may have a synergistic promoting effect on tumorigenesis and development (**Figure 3A**). There was also a significant positive correlation among the 14 lipid metabolism pathways, and all pathways were associated with poor prognosis in AML patients (hazard ratio > 1) (**Figure 3B**), with 9 pathways, including fatty acid degradation, steroid biosynthesis, and primary bile acid biosynthesis, reaching statistical significance (P < 0.05) (**Figure 3C**). On the basis of the activity scores of lipid metabolism pathways, we subsequently performed consensus clustering analysis on AML patients. When k ≥ 3, the CDF distribution becomes flatter. However, the improvement in the area under the CDF curve when k = 4 is not as significant as when k = 3. Therefore, the clustering results when k = 3 are considered (**Figure 3D**). The analysis results indicated that the distinction among the three subtypes (Clusters C1, C2, and C3) was the clearest when patients were divided into three subtypes (**Figure 3D**). The clustering results were verified by the PCA algorithm, which demonstrated high stability and reliability (**Figure 3E**). Among the three subtypes, the activity levels of various lipid metabolism pathways were the highest in the C3 subtype, followed by the C2 subtype, and the lowest activity was detected in the C1 subtype (**Figure 3F**). Notably, the activities of fatty acid metabolism and unsaturated fatty acid biosynthesis pathways were particularly significant in the C3 subtype. Survival analysis revealed that patients in the C1 subtype had the best prognosis, followed by those in the C2 subtype, and those in the C3 subtype had the worst prognosis (**Figure 3G**), suggesting that more active lipid metabolism may promote the malignant proliferation and treatment resistance of leukemia cells, thereby adversely affecting the clinical outcome of patients.

### Analysis of the biological characteristics of lipid metabolism-related molecular subtypes

**Figure 4:**
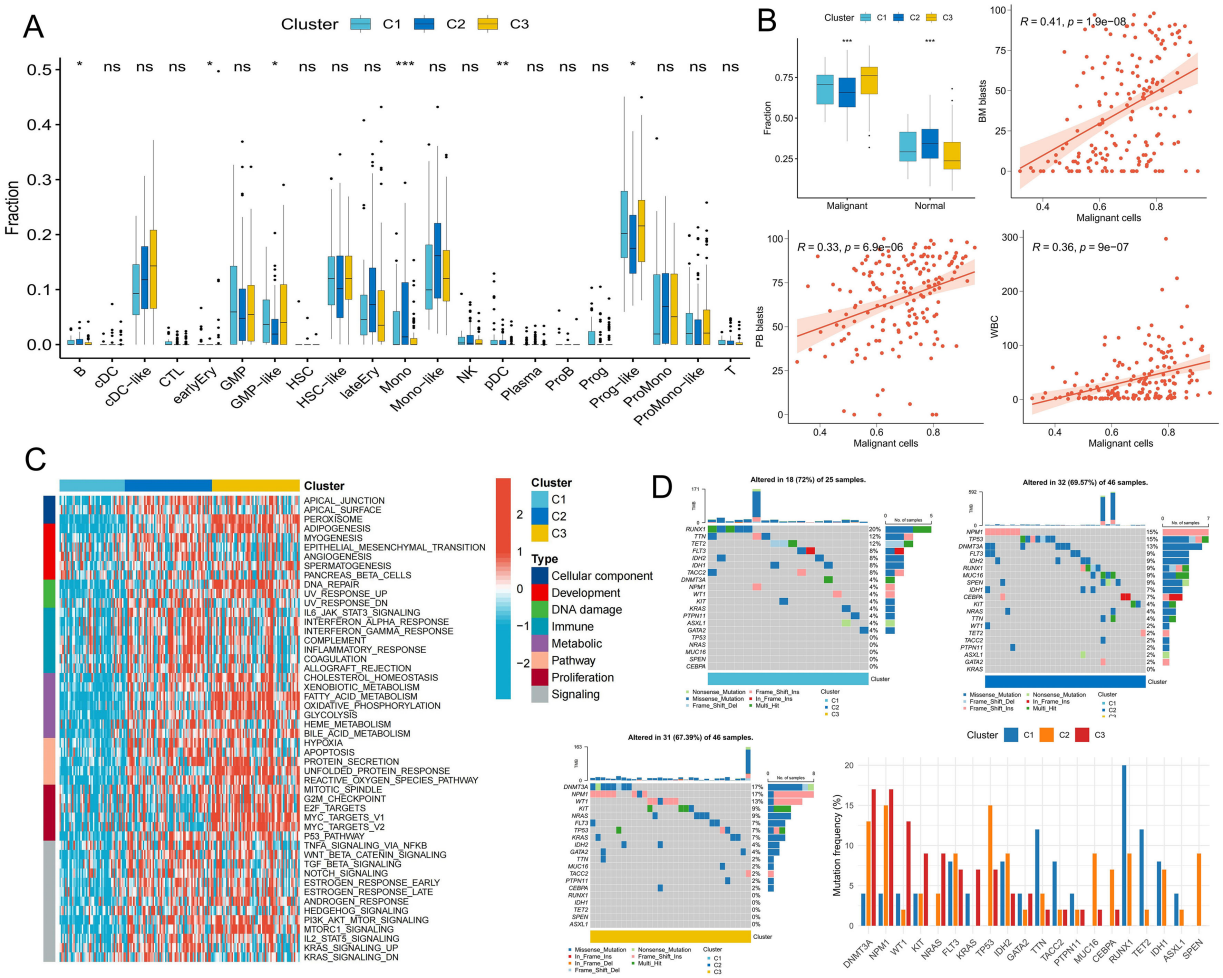
Comparison of the biological characteristics of different lipid metabolism molecular subtypes. **(A, B)** Differences in immune cell infiltration levels among each molecular subtype **(A)** and correlation analysis with peripheral blood blasts (PB blasts), bone marrow blasts (BM blasts), and white blood cell counts (WBCs) **(B)**. **(C, D)** Differences in the activity of tumor hallmark pathways (The darker the red color, the higher the pathway enrichment score; the darker the blue color, the lower the pathway enrichment score.) **(C)** and somatic mutation spectrum features **(D)** among molecular subtypes. (ns, no significance; *P<0.05; **P<0.01; ***P<0.001).

We analyzed the biological characteristics of different lipid metabolism-related subtypes. The immune infiltration analysis results revealed that the proportions of cDC-like, GMP-like, and Prog-like cells were relatively greater in the C3 subtype than in the other subtypes and that the overall proportion of malignant cells significantly increased, whereas the proportion of normal cells significantly decreased (**Figure 4A**). Combined with existing clinical data, the proportion of malignant cells was significantly positively correlated with bone marrow (BM) blasts, peripheral blood (PB) blasts, and white blood cell (WBC) counts, which, to some extent, verified the reliability of the immune infiltration analysis results (**Figure 4B**). The analysis of the activity of cancer hallmark pathways revealed that the activity scores of various pathways were the lowest in the C1 subtype, whereas in the C3 subtype, not only did the activity of the fatty acid metabolism pathway increase but also the activities of oxidative phosphorylation, glycolysis, DNA repair, and multiple proliferation-related pathways significantly increased, further confirming that the C3 subtype has the characteristics of metabolic disorders and active cell proliferation (**Figure 4C**). The three subtypes also show heterogeneity in somatic mutation characteristics: by comparing the three subtypes, RUNX1, TTN and TET2 have the highest mutation frequencies in the C1 subtype, NPM1, MUC16 and SPEN have the highest mutation frequencies in the C2 subtype, and DNMT3A, NPM1 and WT1 have the highest mutation frequencies in the C3 subtype (**Figure 4D**). In addition, we analyzed the incidence of common AML fusion genes. As shown in **Supplementary Figure 2**, no fusion gene was significantly enriched in any specific subtype (Fisher’s exact test, all p > 0.05). These results suggest that the classification based on lipid metabolism is largely independent of these typical oncogenic fusion events.

### Validation of lipid metabolism-related molecular subtypes

**Figure 5:**
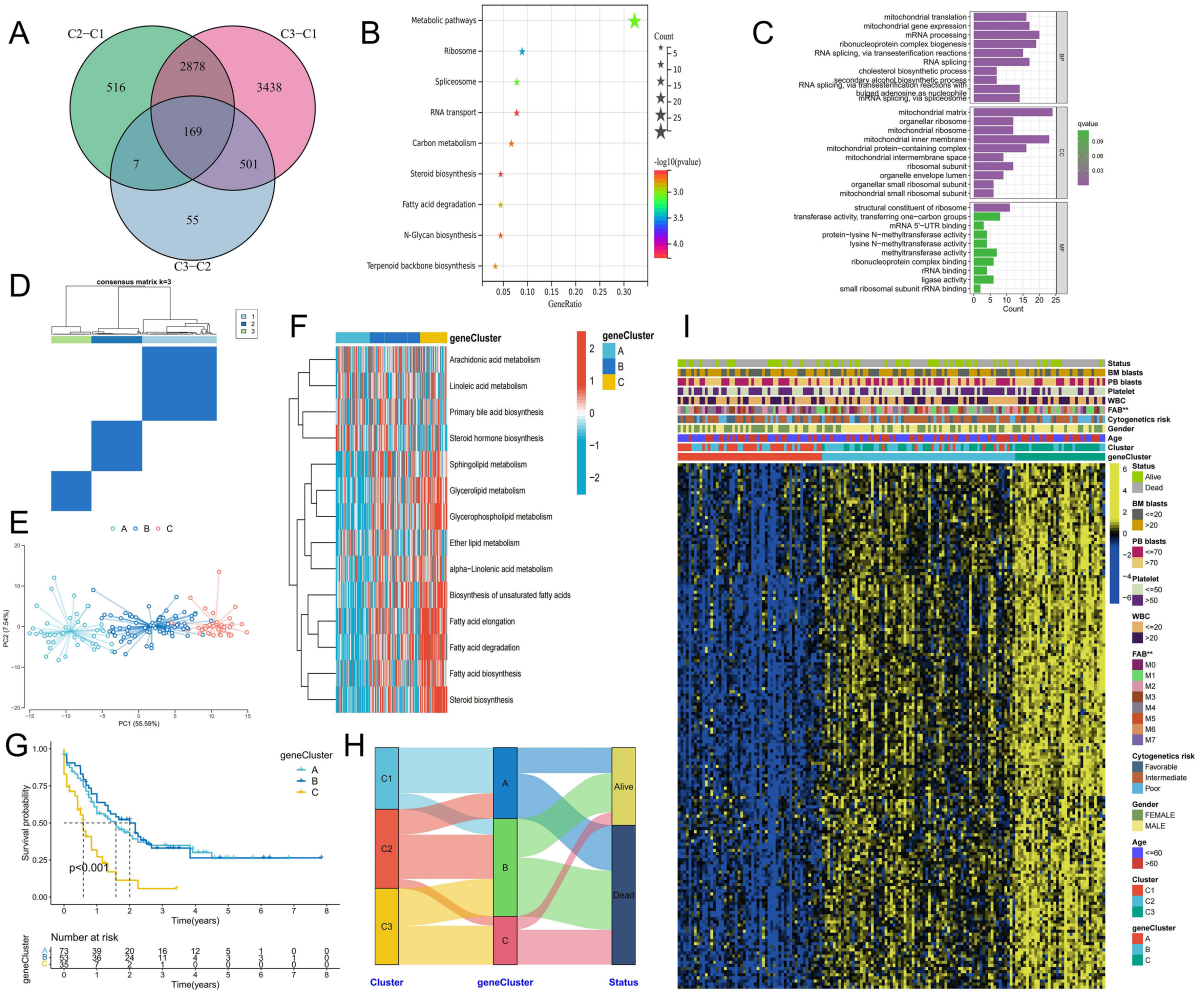
Identification of lipid metabolism-related gene subtypes. **(A–C)** Screening of differentially expressed genes (DEGs) among molecular subtypes **(A)**, KEGG pathway enrichment analysis of DEGs **(B)**, and GO functional annotation **(C)**. **(D)** Division of lipid metabolism-related gene subtypes on the basis of the expression patterns of differentially expressed genes via consensus clustering. **(E)** Evaluation of the separability and robustness of gene subtype clustering through principal component analysis (PCA). **(F, G)** Differences in lipid metabolism scores (The darker the red color, the higher the pathway enrichment score; the darker the blue color, the lower the pathway enrichment score.) **(F)** and patient prognosis **(G)** among different gene subtypes. **(H)** Correspondence between molecular subtypes, gene subtypes, and patient survival status. **(I)** Comparison of clinicopathological features and expression levels of differentially expressed genes among different gene subtypes.

To further validate the existence of lipid metabolism-related molecular subtypes, we conducted differential expression analysis between subtypes and identified 169 significantly differentially expressed genes (**Figure 5A**, **Supplementary Table 3**). Functional enrichment analysis indicated that these genes were enriched mainly in metabolic pathways and were related to functions such as ribosome, spliceosome, and RNA transport (**Figure 5B**). GO annotation analysis further revealed that the functions of these genes involved mainly mitochondrial gene expression, mRNA expression, the mitochondrial matrix, and the structural constituent of the ribosome, suggesting their close association with mitochondrial metabolism and ribosome translation processes (**Figure 5C**). On the basis of the genes with prognostic significance, we performed a reclustering analysis and identified three gene subtypes (geneClusters A, B, and C) with significant discrimination (**Figure 5D**). The stability of the clustering results was verified via the PCA algorithm (**Figure 5E**). The results revealed that lipid metabolism pathway activity was the lowest in gene subtype A and the highest in gene subtype C, and the activity of the fatty acid metabolism pathway was significantly increased (**Figure 5F**). Survival analysis further confirmed that patients with gene subtype C had the poorest prognosis (**Figure 5G**). Sankey diagram analysis revealed that gene subtype A mainly originated from subtype C1, approximately half of gene subtype B originated from C2, and the other half originated from C3, whereas gene subtype C almost entirely originated from subtype C3 (**Figure 5H**). Additionally, the overall expression level of subtype-related genes was the lowest in gene subtype A and the highest in gene subtype C (**Figure 5I**). In conclusion, these results consistently support the existence of three lipid metabolism subtypes in AML patients, with the low lipid metabolism activity subtype having the best prognosis and the high lipid metabolism activity subtype represented by fatty acid metabolism having the worst prognosis.

### Construction of a scoring system for quantifying lipid metabolism-related molecular subtypes

**Figure 6:**
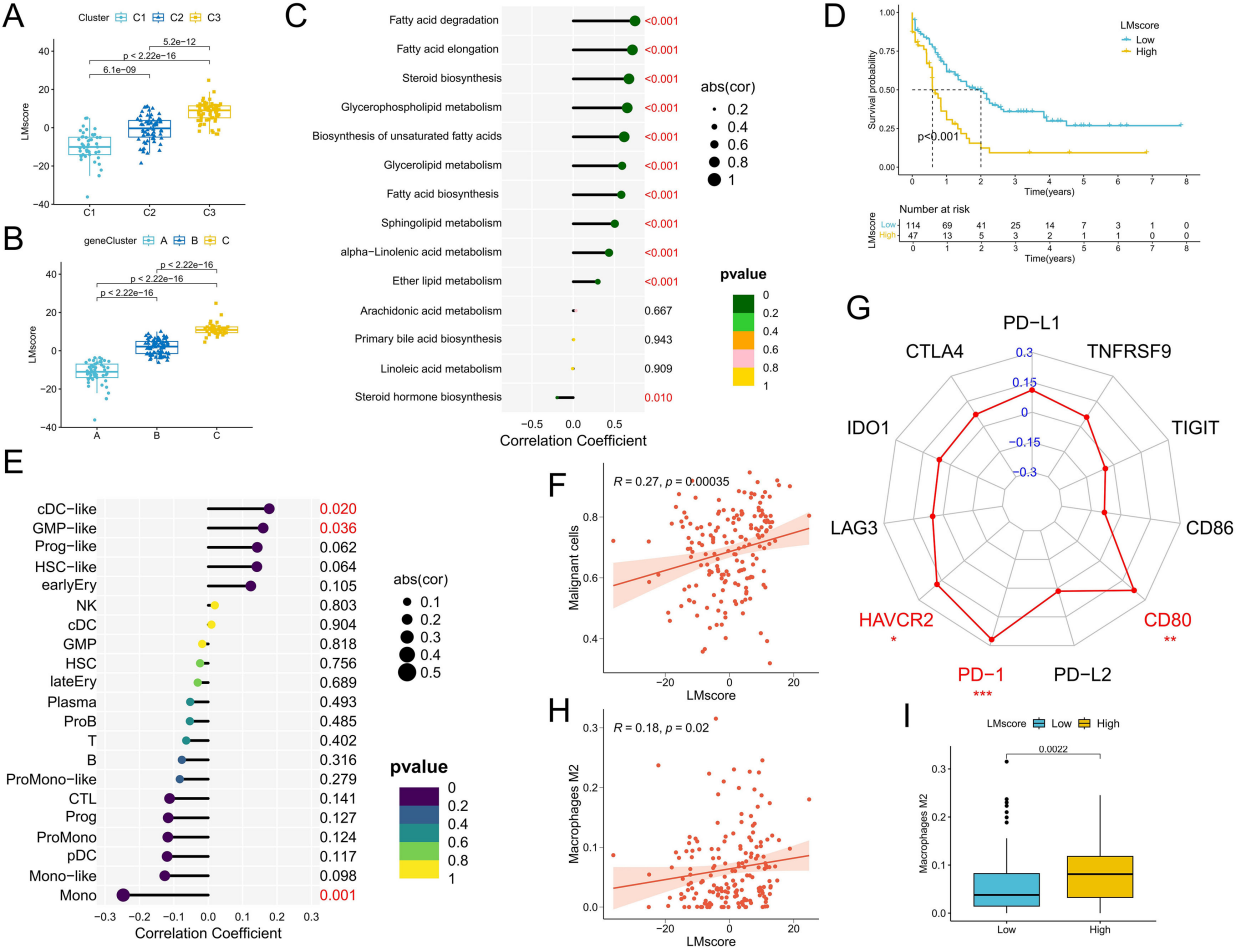
Construction and validation of the LMscore. **(A, B)** Differences in the LMscore among different molecular subtypes and gene subtypes. **(C)** Correlation analysis between the LMscore and lipid metabolism pathway score. **(D)** Differences in survival prognosis between the high and low LMscore groups. **(E–H)** Correlation analysis between the LMscore and the AML cell proportion **(E)**, malignant cell proportion **(F)**, immune checkpoint gene expression level **(G)**, and M2 macrophage infiltration proportion **(H)**. **(I)** Further compare the differences in the infiltration proportion of M2 macrophages among different LMscore groups. (*P<0.05; **P<0.01; ***P<0.001).

To achieve a quantitative assessment of lipid metabolism activity in molecular subtypes, we developed a lipid metabolism scoring system based on the expression data of subtype-related genes via the PCA algorithm and named it the LMscore. The analysis results revealed that the LMscore was the lowest in the C1 subtype, followed by the C2 subtype, and the highest in the C3 subtype (**Figure 6A**); the LMscore was the lowest in gene subtype A, increased in gene subtype B, and was the highest in gene subtype C (**Figure 6B**). Correlation analysis indicated that the LMscore was significantly positively correlated with the scores of most lipid metabolism pathways, including fatty acid metabolism (**Figure 6C**). Survival analysis further demonstrated that patients with high LMscores had significantly poorer prognoses than those with low LMscores (**Figure 6D**), suggesting that this scoring system has good quantitative predictive ability. Further analysis revealed that the LMscore was negatively correlated with the proportions of immune killer cells, such as Mono, pDCs, and CTL cells, but positively correlated with the proportions of AML malignant cells, such as Prog-like, cDC-like, GMP-like, and HSC-like cells (**Figures 6E, F**). This result is consistent with the previously observed active lipid metabolism in AML malignant cells. Additionally, the LMscore was significantly positively correlated with the expression levels of immune checkpoint genes such as HAVCR2, PD-1, and CD80 (**Figure 6G**). The infiltration proportions of immunosuppressive cells, such as M2 macrophages (**Figures 6H, I**), were significantly increased in patients with high LMscores, suggesting that these cells may form an immunosuppressive microenvironment, thereby promoting the immune escape of AML cells. Finally, we validated the gene subtypes and LMscores in two independent cohorts, the Beat AML cohort and the GSE14468 cohort. The results showed that both cohorts could effectively identify gene subtypes with significant differences, and the subtype gene expression and LMscore were the lowest in gene subtype A and the highest in gene subtype C, further verifying the reliability of the results (**Supplementary Figures 3A–H**).

### Construction and validation of a lipid metabolism-related prognostic signature

**Figure 7:**
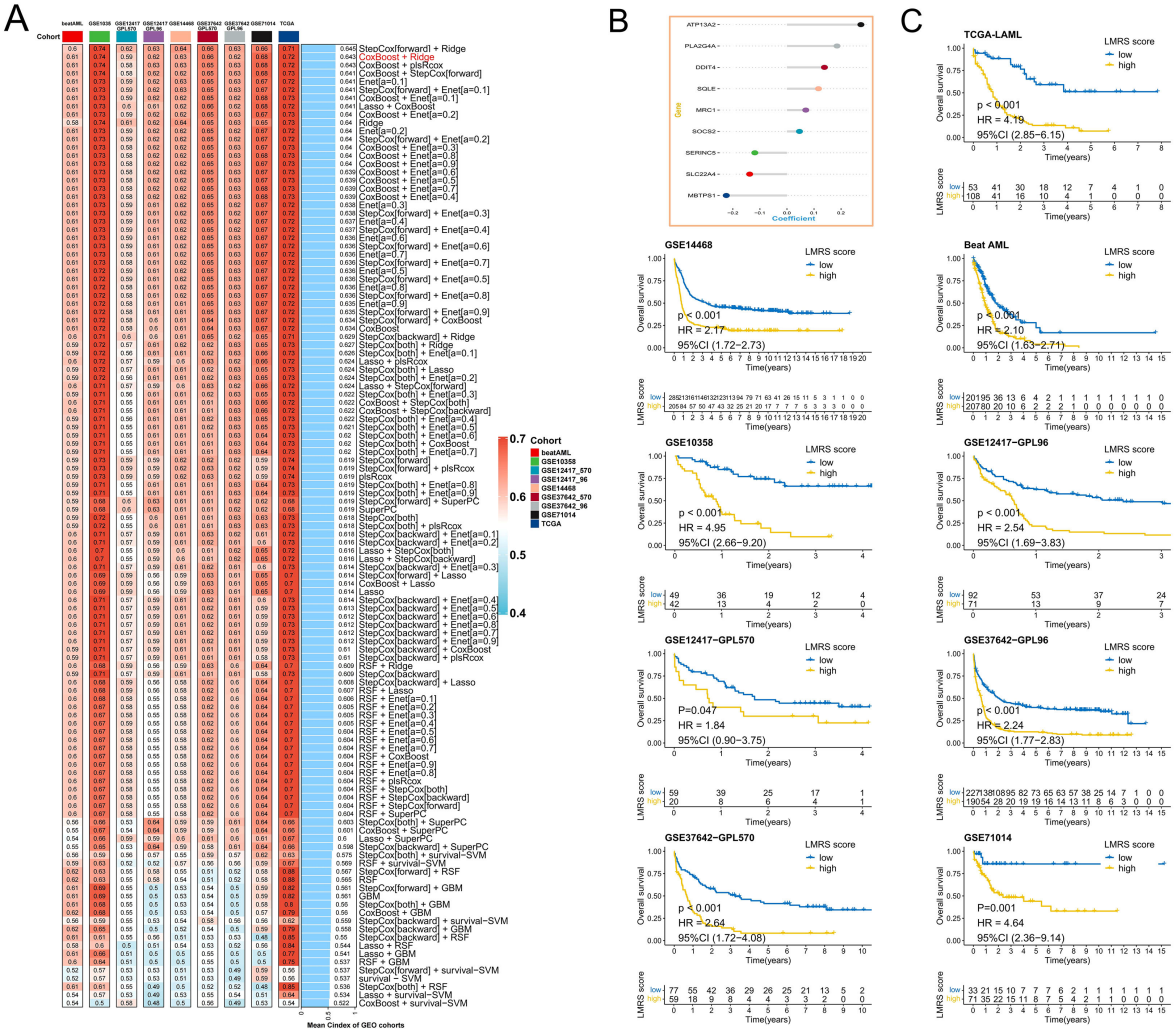
Construction of prognostic models via machine learning. **(A)** Integration of 10 machine learning algorithms generated 117 model combinations. The C-index of each model was calculated in 10 independent cohorts and ranked according to the average C-index of the validation cohort. **(B)** The regression coefficients of each gene in the Ridge model. **(C)** Patients in the 9 AML cohorts were divided into a high LMRS score group and a low LMRS score group according to the best cutoff value, and survival analysis was performed.

To further evaluate the prognostic predictive value of LMRGs, we conducted univariate Cox regression analysis in nine AML cohorts and identified 23 LMRGs significantly associated with the prognosis of AML patients. We subsequently incorporated these LMRGs into an integrated modeling framework to construct a lipid metabolism-related prognostic signature (LMRS). In the TCGA training cohort, we built prognostic models on the basis of 117 algorithm combinations and calculated the C-index of each model and the average C-index of the validation cohort to assess their predictive performance (**Figure 7A**). The results showed that the models combining stepwise Cox (forward direction) with ridge regression (including 23 LMRGs) and CoxBoost with ridge regression (including 9 LMRGs) had the highest average C-index. Among them, the CoxBoost and ridge combination model, which included only 9 genes, had excellent predictive performance and was thus selected as the final model with high accuracy and clinical translational potential. This model consisted of the key LMRGs identified by CoxBoost and the optimal model constructed by Ridge, including 9 core genes: ATP13A2, PLA2G4A, DDIT4, SQLE, MRC1, SOCS2, SERINC5, SLC22A4, and MBTPS1 **Figure 7B**; **Supplementary Figure 4**; **Supplementary Table 2**). We further calculated the LMRS score for each sample in all cohorts, and the results revealed that patients with high LMRS scores exhibited poorer clinical outcomes in all nine AML cohorts (P < 0.05) (**Figure 7C**).

### Prognostic prediction performance evaluation of the LMRS

**Figure 8:**
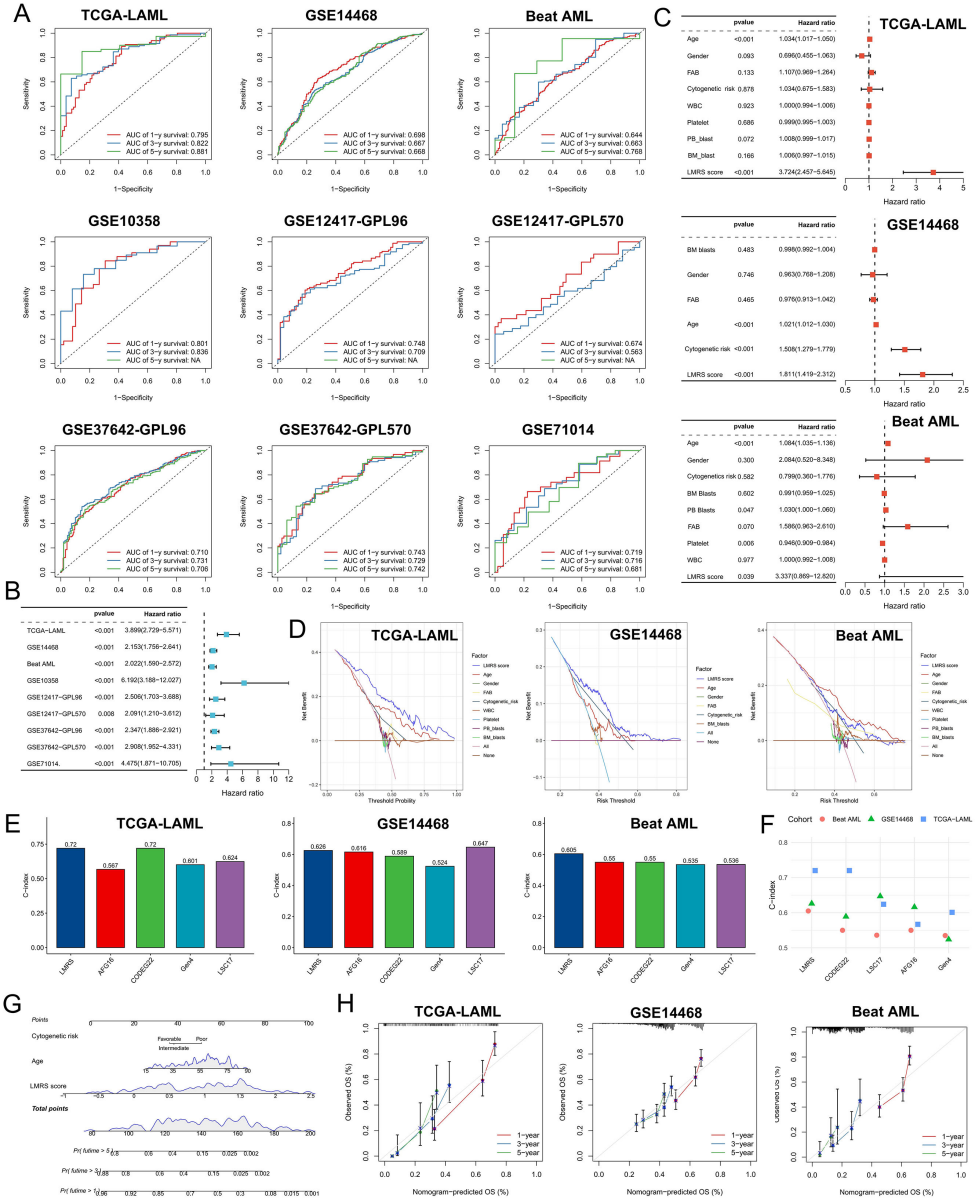
Validation of the prognostic ability of the LMRS score model. **(A)** Time–dependent receiver operating characteristic (ROC) curve analysis of the LMRS model in 9 AML cohorts. **(B)** Univariate Cox regression analysis of the LMRS model in 9 cohorts. **(C, D)** Multivariate Cox regression analysis **(C)** and decision curve analysis (DCA) **(D)** of clinicopathological factors and the LMRS model in the TCGA-LAML, GSE14468, and Beat AML cohorts. **(E, F)** Comparison of the C-index of the LMRS model and existing prognostic models in the above three cohorts. **(G)** Construction of a nomogram combining clinicopathological variables and the LMRS score for predicting the overall survival (OS) of AML patients. **(H)** Calibration curve to evaluate the accuracy of the nomogram in predicting OS.

We further evaluated the prognostic discrimination ability of the LMRS. ROC curve analysis revealed that in the TCGA cohort, the AUC values of the LMRS for predicting the 1-year, 3-year, and 5-year OS of AML patients were 0.795, 0.822, and 0.881, respectively, demonstrating strong predictive performance (**Figure 8A**). In multiple validation cohorts, including GSE14468, Beat AML, GSE37642-GPL96, GSE37642-GPL570, and GSE71014, the LMRS also exhibited good predictive ability, with AUC values generally above 0.65, indicating good stability and generalizability (**Figure 8A**). Additionally, univariate Cox analysis confirmed that LMRS was an important risk factor for OS in AML patients in 9 cohorts (HR > 1, P < 0.01) (**Figure 8B**). After further inclusion of multiple clinical features in multivariate Cox analysis, the results revealed that LMRS was an independent risk factor for OS in the TCGA, GSE14468, and Beat AML cohorts (TCGA-LAML: HR = 3.724 [95% CI: 2.457—5.645], P < 0.001; GSE14468: HR = 1.811 [95% CI: 1.419—2.312], P < 0.001; Beat AML: HR = 3.337 [95% CI: 0.869—12.820], P < 0.05) (**Figure 8C**), further validating its robustness in different datasets. Moreover, decision curve analysis (DCA) results indicated that the LMRS had greater net benefit than other clinical features in clinical decision-making, demonstrating stronger clinical application value (**Figure 8D**). In recent years, several AML prognostic signatures, such as AFG16 (16), CODEG22 (17), Gene4 (18), and LSC17 (19), have been proposed on the basis of transcriptome data. We compared the predictive performance of the LMRS model with those of other models in the TCGA-LAML, GSE14468, and Beat AML cohorts via the C-index. The results showed that the LMRS exhibited superior predictive accuracy in almost all cohorts, further validating its robustness (**Figures 8E, F**). To enhance the clinical utility of the LMRS, we combined the LMRS score with clinical features significantly associated with the prognosis of AML patients (age and cytogenetic risk) to construct a nomogram model (**Figure 8G**). Calibration curve analysis revealed that the predicted results of the nomogram were in good agreement with the actual observed results, indicating its high predictive accuracy (**Figure 8H**).

### The LMRS demonstrated excellent performance in predicting the response to chemotherapy and immunotherapy

**Figure 9:**
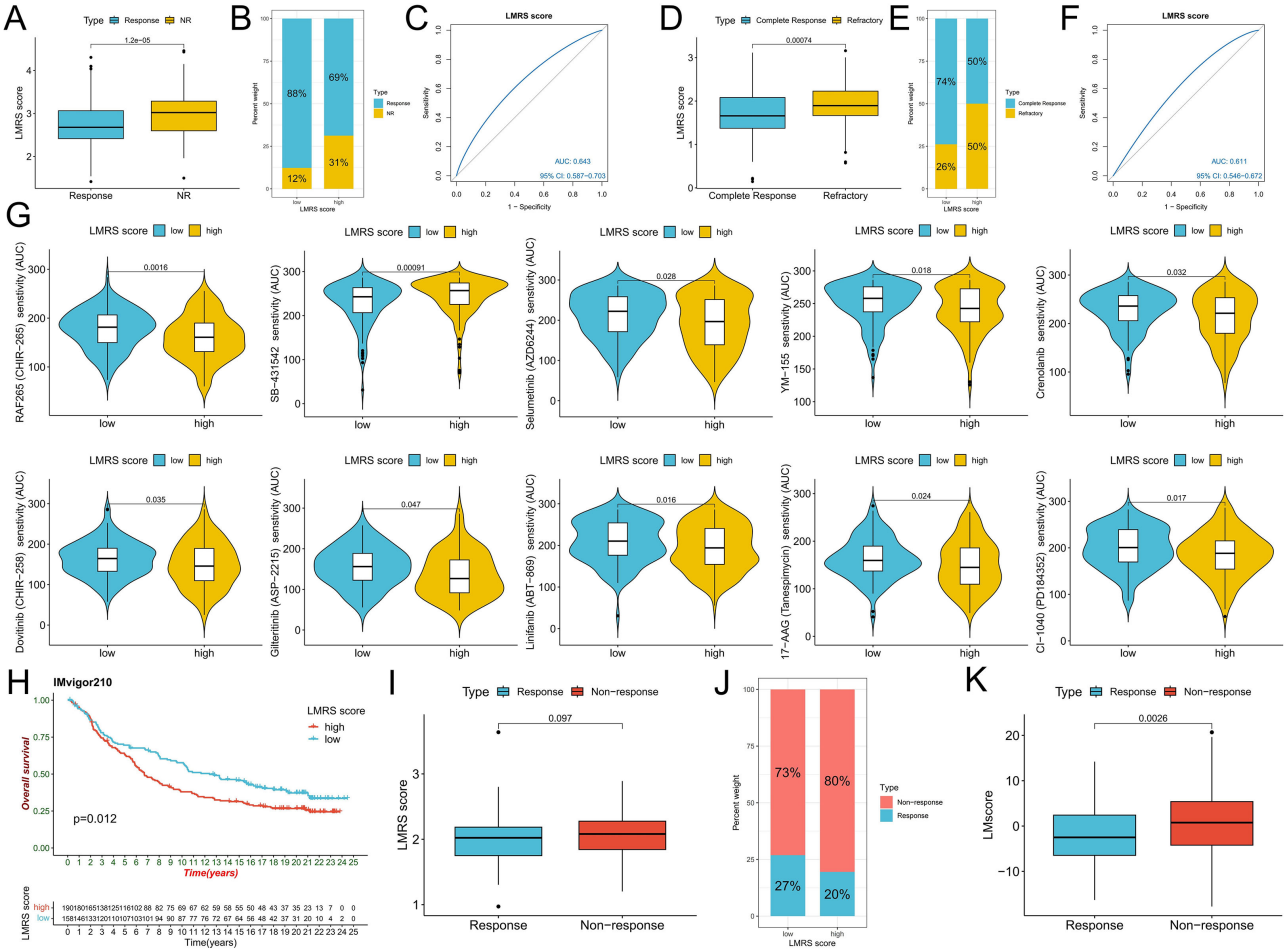
The application value of the LMRS in predicting treatment response. **(A–C)** Differences in the LMRS score between chemotherapy responders and nonresponders in the GSE14468 cohort **(A)**, the distribution of the proportions of the two types of patients in the high and low LMRS score groups **(B)**, and the ROC curve for evaluating the ability of the LMRS score to predict the response to chemotherapy **(C)**. **(D–F)** Relationships between chemotherapy response and the LMRS score in the Beat AML cohort: score differences among patients **(D)**, response proportions in the groups **(E)**, and ROC analysis **(F)**. **(G)** Comparison of sensitivity to multiple chemotherapy drugs between the high- and low-LMRS score groups. **(H–K)** Survival analysis of the high and low LMRS score groups in the IMvigor210 cohort **(H)**, differences in the LMRS score between immune therapy responders and nonresponders **(I)**, comparisons of the response proportions between the two groups **(J)**, and differences in the LMRS score according to immune therapy response status **(K)**.

To comprehensively evaluate the predictive value of LMRS in the treatment response of AML patients, we conducted an analysis in the GSE14468 and Beat AML cohorts, which included data on the response to chemotherapy. The results revealed that, in both cohorts, the LMRS score of patients who responded to chemotherapy was significantly lower than that of nonresponders (**Figures 9A, D**), and the proportion of nonresponders was significantly greater in the high LMRS score group (**Figures 9B, E**). ROC curve analysis revealed that the AUC values of the LMRS for predicting chemotherapy response in the GSE14468 and Beat AML cohorts were 0.643 and 0.611, respectively, indicating modest discriminatory ability, suggesting that LMRS is exploratory and hypothesis−generating for chemotherapy response prediction (**Figures C, F**). The Beat AML cohort also provided *in vitro* response data of AML cells to multiple drugs. The analysis revealed that patients with high LMRS scores were more sensitive to RAF265, selumetinib, YM-155, crenolanib, dovitinib, gilteritinib, linifanib, 17-AAG, and CI-1040, whereas patients with low LMRS scores were more sensitive to SB-431542, suggesting that these drugs may have potential therapeutic value for patients with different LMRS scores (**Figure 9G**). To further explore the potential of LMRS in predicting immunotherapy response in a non-AML context, we included the IMvigor210 cohort (atezolizumab-treated urothelial carcinoma) as an external validation. The results revealed that patients in the high LMRS score group had a significantly worse prognosis and a greater proportion of nonresponders (**Figure 9H**); at the same time, the LMRS score of nonresponders was significantly greater than that of responders (**Figures 9I–J**), and the LMscore showed a similar trend (**Figure 9J**). In conclusion, these results support the potential value of the LMRS in predicting the efficacy of immunotherapy, suggesting that patients with high LMRS scores may benefit more from immunotherapy.

### Identification and functional analysis of key LMRGs

**Figure 10:**
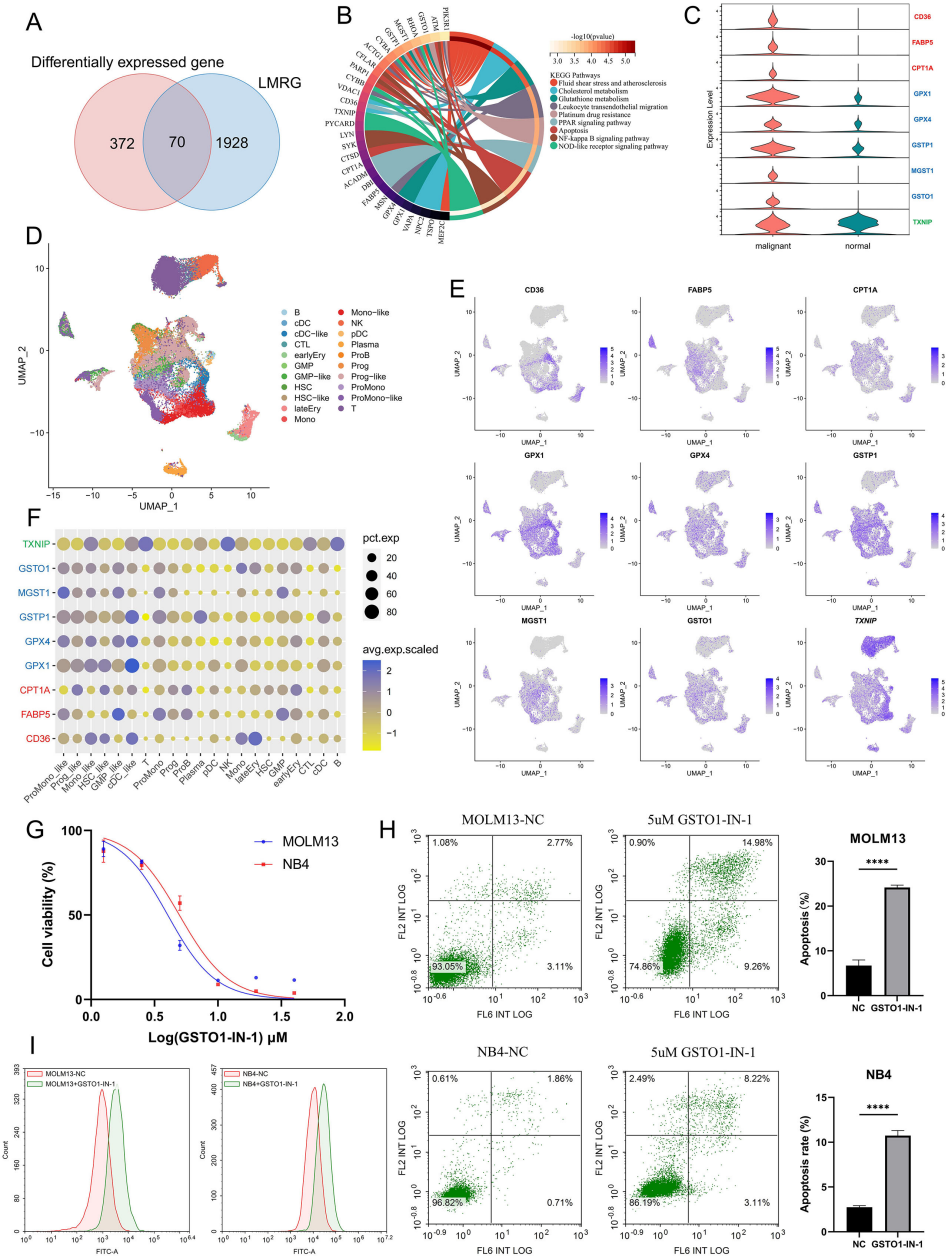
Identification and functional validation of key lipid metabolism-related genes (LMRGs). **(A)** Intersection analysis of genes differentially expressed between AML malignant cells and normal cells with LMRGs. **(B)** KEGG pathway enrichment results of the intersection genes. **(C)** Differences in the expression levels of key LMRGs in AML malignant cells and normal cells. **(D)** Cell type annotation results of single-cell data. **(E, F)** Expression distribution characteristics of key LMRGs at the single-cell level. **(G)** A CCK8 assay was used to detect changes in the viability of MOLM13 and NB4 cells treated with different concentrations of GSTO1-IN-1. **(H)** Flow cytometry was used to detect differences in the number of apoptotic MOLM13 and NB4 cells treated with 5 μM GSTO1-IN-1 compared with the normal control group (NC). **(I)** Changes in intracellular reactive oxygen species (ROS) levels under the same conditions. (****P<0.0001).

To further reveal the key LMRGs in AML cells, we conducted an intersection analysis of differentially expressed genes between AML malignant cells and normal cells in single-cell data, identifying a total of 70 LMRGs that were significantly differentially expressed in AML malignant cells (**Figure 10A**). The functional enrichment analysis revealed that these genes were enriched in pathways such as fluid shear stress and atherosclerosis, cholesterol metabolism, glutathione metabolism, leukocyte transendothelial migration, platinum drug resistance, the PPAR signaling pathway, apoptosis, the NF-kappa B signaling pathway, and the NOD-like receptor signaling pathway (**Figure 10B**). Through single-cell data analysis, we found that genes involved in fatty acid transport and metabolism (such as CD36, FABP5, and CPT1A) and genes involved in glutathione metabolism (such as GPX1, GPX4, GSTP1, MGSTP1, and GSTO1) were significantly upregulated in AML malignant cells (**Figures 10C–F**). Among them, glutathione metabolism mediated by genes such as GPX4 has an antioxidant stress effect and can inhibit the generation of reactive oxygen species (ROS). Moreover, we observed that the gene encoding TXNIP, which promotes oxidative stress, was downregulated in AML malignant cells. These results suggest that the malignant proliferation of AML cells may depend on the ROS detoxification mechanism mediated by fatty acid metabolism and glutathione metabolism. Therefore, we speculate that inhibiting the expression of glutathione metabolism-related genes to promote ROS generation may help inhibit the growth of AML cells. Given that the functions of GPX1, GPX4 and GSTP1 have been widely reported in numerous AML studies (20–22), this research focused on GSTO1, which is significantly differentially expressed between AML cells and normal cells. The AML cell lines NB4 and MOLM13 were treated with the GSTO1-specific inhibitor GSTO1-IN-1. The CCK8 assay results indicated that GSTO1-IN-1 could effectively inhibit the proliferation of AML cells, with IC50 values of 5.526 μM in NB4 cells and 3.968 μM in MOLM13 cells (**Figure 10G**). Further experiments demonstrated that, after treatment with 5 μM GSTO1-IN-1, the apoptosis rates of NB4 and MOLM13 cells were significantly greater than those of the control group (**Figure 10H**), and the intracellular ROS levels also increased significantly (**Figure 10I**). These results suggest that GSTO1 is a candidate vulnerability in AML cell lines, warranting further investigation in preclinical models and primary samples before clinical translation.

## Discussion

AML is a hematological malignancy with high molecular and clinical heterogeneity, and its prognosis remains unsatisfactory (23). In recent years, the role of metabolic reprogramming, especially aerobic glycolysis (the Warburg effect), in tumorigenesis and development has been widely recognized (24, 25). However, systematic studies on lipid metabolism in AML and its clinical significance still need to be conducted. In this study, by integrating multiomics data and *in vitro* experiments, we systematically revealed the key role of lipid metabolism in AML and constructed a powerful lipid metabolism-related prognostic signature (LMRS), providing new insights for the precise classification and treatment of AML.

This study is the first to map lipid metabolism in AML at the single-cell level. Compared with normal cells, the malignant AML cells, especially progenitor cell subpopulations such as ProMono-like, Prog-like and GMP-like cells, presented significantly upregulated activities in multiple lipid metabolism pathways, including fatty acid metabolism and glycerophospholipid metabolism. This finding is consistent with the classic paradigm in solid tumors that “lipid metabolism reprogramming promotes rapid tumor growth” (26), indicating that AML cells increase lipid uptake and catabolism to meet the energy, biomembrane construction and signal molecule precursor requirements for their rapid proliferation. Subsequent deconvolution analysis further confirmed at the population level that the proportion of malignant cells represented by cDC-like and GMP-like cells was significantly positively correlated with lipid metabolism activity, suggesting that tumor cells themselves are the main source of abnormal lipid metabolism.

Consensus clustering based on lipid metabolism pathways successfully classified AML patients into three molecular subtypes (C1-C3) with significant prognostic differences. Among them, the C3 subtype exhibited the highest lipid metabolism activity and the poorest clinical prognosis, and its cancer hallmark pathways (such as proliferation, DNA repair, and oxidative phosphorylation) were comprehensively enhanced, presenting an aggressive tumor biological image of “high metabolism and high proliferation”. This result strongly suggests that the hyperactivity of lipid metabolism is not an isolated event but is closely coupled with the overall malignant progression of AML, possibly driving the aggressiveness and treatment resistance of the disease by providing energy and biomacromolecules.

The poor prognosis of the C3 subtype may also be related to the immunosuppressive microenvironment. We developed the LMscore system to quantify the three molecular subtypes, among which the C1 subtype has the lowest LMscore and the C3 subtype has the highest. Our analysis revealed that a high LMscore is significantly associated with a reduction in the infiltration of immune killer cells (such as CTLs), an increase in the infiltration of immunosuppressive cells (such as M2 macrophages), and the upregulation of multiple immune checkpoint molecules (such as HAVCR2 and PD-1). These findings indicate that AML with active lipid metabolism may shape a “cold” tumor microenvironment, thereby promoting immune escape. These findings provide a theoretical basis for the therapeutic strategy of combining lipid metabolism regulation with immune checkpoint inhibitors.

To translate this biological discovery into clinical application potential, we constructed a concise yet powerful lipid metabolism-related prognostic signature (LMRS). The LMRS comprises nine genes with diverse functions that can be linked to lipid metabolism and AML pathogenesis. For instance, SQLE (squalene epoxidase) is a rate-limiting enzyme in cholesterol biosynthesis, and its upregulation is linked to poor prognosis in various cancers (27). PLA2G4A (phospholipase A2 group IVA) is crucial for the release of arachidonic acid from membranes, fueling inflammatory and survival signaling pathways (28). DDIT4 (DNA damage inducible transcript 4) is a stress-response gene that regulates mTOR signaling, a central pathway integrating nutrient and growth factor signals, including those from lipids (29). MRC1 (mannose receptor C-type 1) is a marker of M2-polarized macrophages, directly linking a high LMRS to the immunosuppressive microenvironment we observed (30). The inclusion of ATP13A2, a lysosomal transporter (31), and SOCS2, a negative regulator of cytokine signaling (32), further highlights the complex interplay between metabolic stress, immune evasion, and leukemic cell survival. In nine independent AML cohorts, LMRS consistently demonstrated excellent prognostic prediction performance. Its predictive ability outperformed multiple previously published signatures (such as LSC17), indicating that the LMRS captures a core biological dimension in the disease progression of AML. More importantly, the LMRS is not only a prognostic indicator but also a potential therapeutic guidance tool. We found that a high LMRS score was significantly associated with chemotherapy resistance and could predict the sensitivity of AML cells to a series of targeted drugs (such as RAF265 and gilteritinib). Notably, in the IMvigor210 immunotherapy cohort, a high LMRS was also associated with poor prognosis and treatment resistance, extending its predictive value to the field of immunotherapy.

By integrating single-cell data, we further identified the key LMRGs that were upregulated in AML malignant cells and focused on the glutathione metabolism pathway. This pathway is a core system for the cellular antioxidant stress response. Our functional experiments confirmed that inhibiting the key gene GSTO1 in this pathway can effectively induce the apoptosis of AML cells and increase ROS levels, thereby suppressing cell proliferation. These findings demonstrate that the survival and proliferation of AML cells are highly dependent on the protective effects provided by lipid and antioxidant metabolism. Targeting these pathways represents a highly promising therapeutic strategy.

This study also has several limitations. Firstly, although retrospective cohort analysis can reveal strong associations, prospective clinical studies are still needed to further validate the clinical utility of the LMRS. Secondly, the specific molecular mechanism of the interaction between lipid metabolism and the immune microenvironment is not yet fully clear and requires further exploration. The immunotherapy response analysis was performed in a non-AML cohort (urothelial carcinoma); thus, the conclusions regarding immunotherapy in AML remain speculative and require direct validation in AML patients receiving immune checkpoint inhibitors. Furthermore, our analysis of somatic mutations was limited to frequently mutated genes in the TCGA cohort and did not include a comprehensive assessment of fusion genes, which are known drivers in AML and should be investigated in relation to lipid metabolism subtypes in future studies with more comprehensive genomic data. Finally, while our *in vitro* data confirm that GSTO1 inhibition induces apoptosis and ROS accumulation in AML cells, we acknowledge that several genes in our signature, such as GPX4, are canonical regulators of ferroptosis—an iron-dependent form of cell death driven by lipid peroxidation. The observed increase in ROS could serve as a trigger for both apoptotic and ferroptotic pathways. However, a detailed dissection of the precise cell death mechanism—whether apoptosis, ferroptosis, or a combination thereof—requires systematic investigation using specific pathway inhibitors and lipid peroxidation assays. As the primary aim of this study was to identify and validate lipid metabolism-related targets from a multi-omics landscape, such in-depth mechanistic exploration falls beyond the current scope and will be addressed in future work.

In summary, this study systematically revealed the core role of lipid metabolism in AML, defined lipid metabolism molecular subtypes with significant clinical significance, and developed a powerful and extensively validated prognostic and predictive model, the LMRS. Our research not only contributes to the understanding of metabolic heterogeneity in AML but also, more importantly, provides new practical tools for risk stratification, prognosis prediction, and treatment strategy selection for AML patients and highlights the value of targeting lipid metabolism pathways (especially glutathione metabolism) as a potential new direction for AML treatment.

## Data Availability

The original contributions presented in the study are included in the article/**Supplementary Material**. Further inquiries can be directed to the corresponding author.

## References

[1] Kantarjian HM DiNardo CD Kadia TM Daver NG Altman JK Stein EM . Acute myeloid leukemia management and research in 2025. CA: A Cancer J For Clin. (2025) 75:46–67. doi: 10.3322/caac.21873 39656142 PMC11745214

[2] Forsberg M Konopleva M . AML treatment: conventional chemotherapy and emerging novel agents. Trends Pharmacol Sci. (2024) 45:430–48. doi: 10.1016/j.tips.2024.03.005 38643058

[3] Nong S Han X Xiang Y Qian Y Wei Y Zhang T . Metabolic reprogramming in cancer: mechanisms and therapeutics. MedComm. (2023) 4:e218. doi: 10.1002/mco2.218 36994237 PMC10041388

[4] Liao M Yao D Wu L Luo C Wang Z Zhang J . Targeting the Warburg effect: a revisited perspective from molecular mechanisms to traditional and innovative therapeutic strategies in cancer. Acta Pharm Sin B. (2024) 14:953–1008. doi: 10.1016/j.apsb.2023.12.003 38487001 PMC10935242

[5] Schiliro C Firestein BL . Mechanisms of metabolic reprogramming in cancer cells supporting enhanced growth and proliferation. Cells. (2021) 10(5):1056. doi: 10.3390/cells10051056 33946927 PMC8146072

[6] Cheng C Geng F Cheng X Guo D . Lipid metabolism reprogramming and its potential targets in cancer. Cancer Commun (London England). (2018) 38:27. doi: 10.1186/s40880-018-0301-4 29784041 PMC5993136

[7] Broadfield LA Pane AA Talebi A Swinnen JV Fendt SM . Lipid metabolism in cancer: new perspectives and emerging mechanisms. Dev Cell. (2021) 56:1363–93. doi: 10.1016/j.devcel.2021.04.013 33945792

[8] Yin X Xu R Song J Ruze R Chen Y Wang C . Lipid metabolism in pancreatic cancer: emerging roles and potential targets. Cancer Commun (London England). (2022) 42:1234–56. doi: 10.1002/cac2.12360 36107801 PMC9759769

[9] Wang X Li Y Hou X Li J Ma X . Lipid metabolism reprogramming in endometrial cancer: biological functions and therapeutic implications. Cell Communication Signaling CCS. (2024) 22:436. doi: 10.1186/s12964-024-01792-7 39256811 PMC11385155

[10] Alannan M Fayyad-Kazan H Trézéguet V Merched A . Targeting lipid metabolism in liver cancer. Biochemistry. (2020) 59:3951–64. doi: 10.1021/acs.biochem.0c00477 32930581

[11] Riaz AA Ginimol M Rasha R Ahmed K Ahmed A Catrin S . Transparency in the reporting of artificial intelligence – the TITAN guideline. Premier J Sci. (2025) 10:100082. doi: 10.70389/pjs.100082

[12] Zhong F Chen S Yao F Liu J Zhang J Jiang J . Machine learning-driven glycolytic subtyping and exosome-based PKM splicing modulation overcome drug resistance in hyper-glycolytic myeloid leukemia. NPJ Digital Med. (2025) 9:18. doi: 10.1038/s41746-025-02185-x 41326769 PMC12780028

[13] Hänzelmann S Castelo R Guinney J . GSVA: gene set variation analysis for microarray and RNA-seq data. BMC Bioinf. (2013) 14:7. doi: 10.1186/1471-2105-14-7 PMC3618321 23323831

[14] Newman A Liu C Green M Gentles A Feng W Xu Y . Robust enumeration of cell subsets from tissue expression profiles. Nat Methods. (2015) 12:453–7. doi: 10.1038/nmeth.3337 25822800 PMC4739640

[15] Wilkerson M Hayes D . ConsensusClusterPlus: a class discovery tool with confidence assessments and item tracking. Bioinf (Oxford England). (2010) 26:1572–3. doi: 10.1093/bioinformatics/btq170 20427518 PMC2881355

[16] Jin P Jin Q Wang X Zhao M Dong F Jiang G . Large-scale *in vitro* and *in vivo* CRISPR-Cas9 knockout screens identify a 16-gene fitness score for improved risk assessment in acute myeloid leukemia. Clin Cancer Res. (2022) 28:4033–44. doi: 10.1158/1078-0432.ccr-22-1618 35877119 PMC9475249

[17] Nehme A Dakik H Picou F Cheok M Preudhomme C Dombret H . Horizontal meta-analysis identifies common deregulated genes across AML subgroups providing a robust prognostic signature. Blood Adv. (2020) 4:5322–35. doi: 10.1182/bloodadvances.2020002042 33108456 PMC7594391

[18] Chen Z Song J Wang W Bai J Zhang Y Shi J . A novel 4-mRNA signature predicts the overall survival in acute myeloid leukemia. Am J Hematol. (2021) 96:1385–95. doi: 10.1002/ajh.26309 34339537

[19] Ng SW Mitchell A Kennedy JA Chen WC McLeod J Ibrahimova N . A 17-gene stemness score for rapid determination of risk in acute leukaemia. Nature. (2016) 540:433–7. doi: 10.1038/nature20598 27926740

[20] Zhang J Peng Y He Y Xiao Y Wang Q Zhao Y . GPX1-associated prognostic signature predicts poor survival in patients with acute myeloid leukemia and involves in immunosuppression. Biochim Biophys Acta Mol Basis Dis. (2022) 1868:166268. doi: 10.1016/j.bbadis.2021.166268 34536536

[21] Akiyama H Zhao R Ostermann LB Li Z Tcheng M Yazdani SJ . Mitochondrial regulation of GPX4 inhibition-mediated ferroptosis in acute myeloid leukemia. Leukemia. (2024) 38:729–40. doi: 10.21203/rs.3.rs-2657913/v1 38148395 PMC11082873

[22] Luo J Ding L Pan S Luo J Zhao H Yin J . SPAG6 overexpression decreases the pro-apoptotic effect of daunorubicin in acute myeloid leukemia cells through the ROS/JNK MAPK axis in a GSTP1-dependent manner. Front Pharmacol. (2024) 15:1390456. doi: 10.3389/fphar.2024.1390456 39508041 PMC11537985

[23] Döhner H Wei A Löwenberg B . Towards precision medicine for AML. Nat Rev Clin Oncol. (2021) 18(9):577–90. doi: 10.1038/s41571-021-00509-w 34006997

[24] Yang Y Pu J Yang Y . Glycolysis and chemoresistance in acute myeloid leukemia. Heliyon. (2024) 10:e35721. doi: 10.1016/j.heliyon.2024.e35721 39170140 PMC11336864

[25] Icard P Shulman S Farhat D Steyaert JM Alifano M Lincet H . How the Warburg effect supports aggressiveness and drug resistance of cancer cells? Drug Resistance Updates Rev Commentaries Antimicrobial Anticancer Chemotherapy. (2018) 38:1–11. doi: 10.1016/j.drup.2018.03.001 29857814

[26] Bian X Liu R Meng Y Xing D Xu D Lu Z . Lipid metabolism and cancer. J Exp Med. (2020) 218(1):e20201606. doi: 10.1084/jem.20201606 33601415 PMC7754673

[27] Xu H Zhou S Tang Q Xia H Bi F . Cholesterol metabolism: new functions and therapeutic approaches in cancer. Biochim Biophys Acta Rev Cancer. (2020) 1874:188394. doi: 10.1016/j.bbcan.2020.188394 32698040

[28] Zhao R Lv Y Feng T Zhang R Ge L Pan J . ATF6α promotes prostate cancer progression by enhancing PLA2G4A-mediated arachidonic acid metabolism and protecting tumor cells against ferroptosis. Prostate. (2022) 82:617–29. doi: 10.1002/pros.24308 35089606 PMC9303695

[29] Dai B Xu L Rong S Song M Lan Z Chen W . YTHDF2 promotes anaplastic thyroid cancer progression by activating the DDIT4/AKT/mTOR signaling pathway. Biol Direct. (2024) 19:122. doi: 10.1186/s13062-024-00566-y 39593172 PMC11600618

[30] Hughes R Qian BZ Rowan C Muthana M Keklikoglou I Olson OC . Perivascular M2 macrophages stimulate tumor relapse after chemotherapy. Cancer Res. (2015) 75:3479–91. doi: 10.1158/0008-5472.can-14-3587 26269531 PMC5024531

[31] van Veen S Martin S Van den Haute C Benoy V Lyons J Vanhoutte R . ATP13A2 deficiency disrupts lysosomal polyamine export. Nature. (2020) 578:419–24. doi: 10.1038/s41586-020-1968-7 31996848

[32] Letellier E Haan S . SOCS2: physiological and pathological functions. Front Bioscience (Elite Edition). (2016) 8:189–204. doi: 10.2741/E760 26709655

