# Supplementary material for: Integration of transcriptomic data reveals lipid metabolic heterogeneity and identifies GSTO1 as a therapeutic target in acute myeloid leukemia
Source: Front Immunol. 2026 Jun 30;17:1815163. doi: 10.3389/fimmu.2026.1815163 (PMC13364964; doi:10.3389/fimmu.2026.1815163)
Supplement: Supplementary file 1 [file Table1.docx]

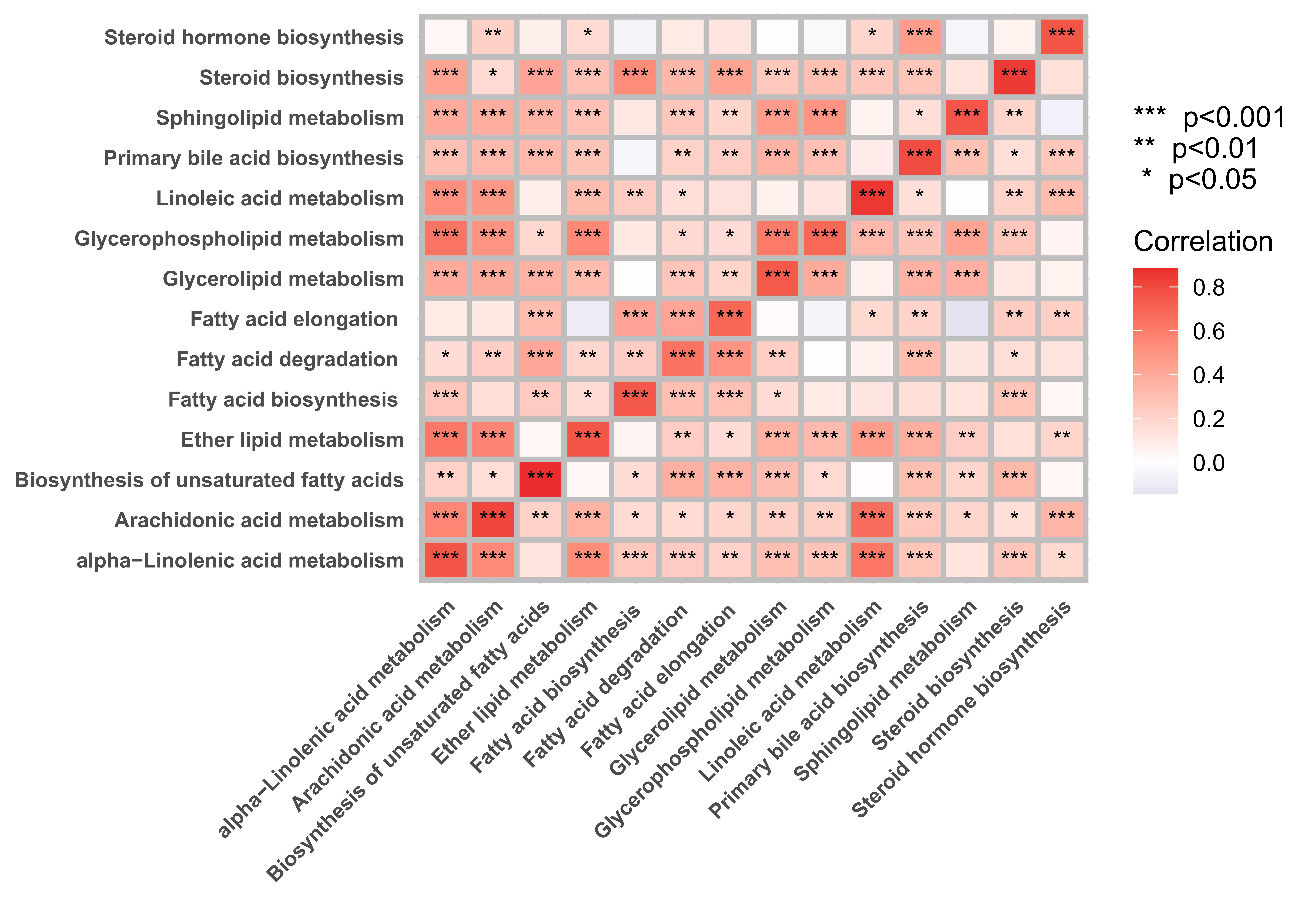


Figure S1. Correlation analysis of ssGSEA scores (x-axis) and GSVA scores (y-axis) of 14 lipid metabolism pathways in the TCGA-LAML cohort. The darker the color, the greater the correlation. (*P<0.05; **P<0.01; ***P<0.001)


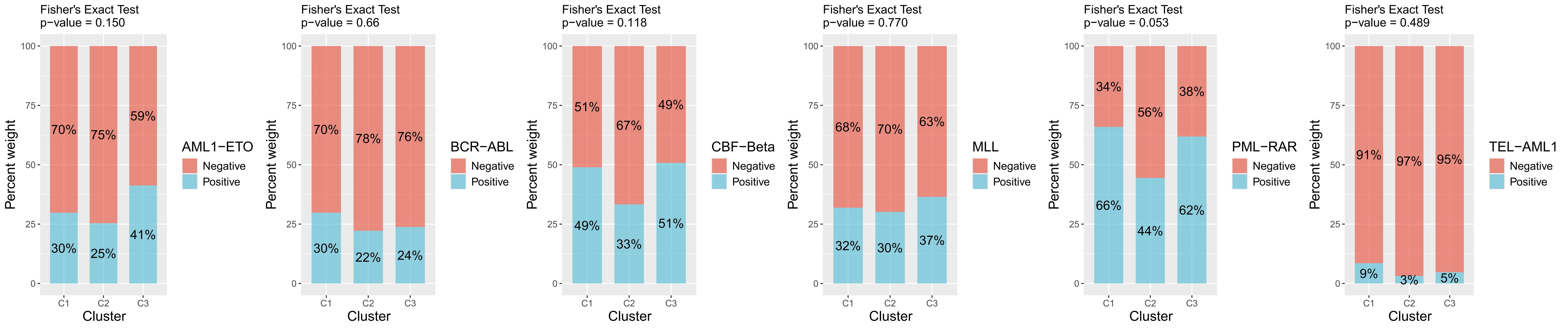


Figure S2. Distribution of common AML fusion genes across the three lipid metabolism subtypes (C1–C3). Bars represent the percentage of patients with the indicated fusion within each subtype. Fisher’s exact test was used to compare fusion prevalence among subtypes; p-values are shown above each bar.





Figure S3. (A-D) In the GSE14468 cohort, consensus clustering was used to classify lipid metabolism-related genes into molecular subtypes (A), and the expression levels of differentially expressed genes (B), lipid metabolism pathway activity scores (C), and LMscore (D) were compared among different subtypes. (E-H) In the Beat AML cohort, consensus clustering was also applied to identify gene subtypes (E), and the expression profiles of differentially expressed genes (F), lipid metabolism pathway scores (G), and LMscore (H) were compared among subtypes.


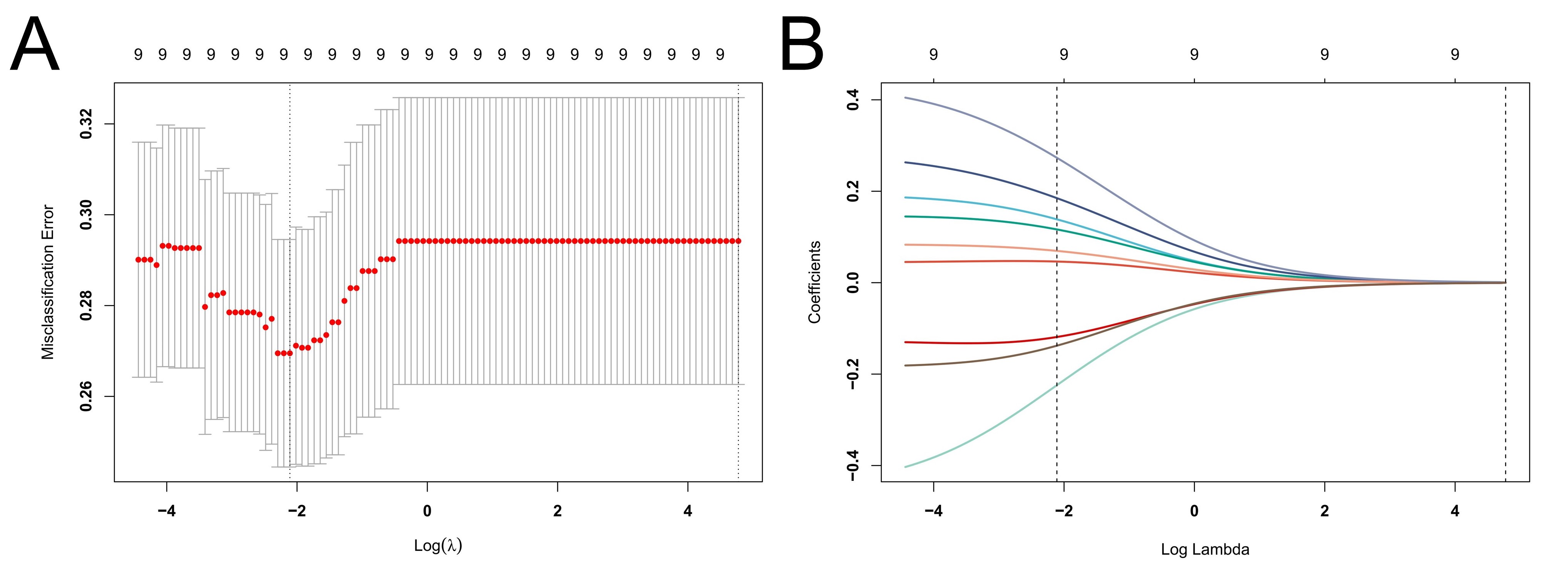


Figure S4. The construction parameters of the Ridge model. (A) The optimal penalty coefficient corresponding to the minimum 10-fold cross-validation error was determined. (B) The regression coefficients of each gene in the final model were estimated on the basis of the selected penalty coefficient.
